# Knowledge, attitude, and readiness towards disaster management: A nationwide survey among healthcare practitioners in United Arab Emirates

**DOI:** 10.1371/journal.pone.0278056

**Published:** 2023-02-16

**Authors:** Sawsan Shanableh, Muaed Jamal Alomar, Subish Palaian, Mohammad Majed Al-Ahmad, Mohamed Izham Mohamed Ibrahim

**Affiliations:** 1 Department of Clinical Sciences, College of Pharmacy and Health Sciences, Ajman University, Ajman, UAE; 2 Department of Clinical Pharmacy, College of Pharmacy, Al Ain University, Al Ain Campus, Al Ain, UAE; 3 Department of Clinical Pharmacy and Practice, College of Pharmacy, QU Health, Qatar University, Doha, Qatar; Quaid-i-Azam University, PAKISTAN

## Abstract

Health professionals are expected to be knowledgeable on disaster medicine and prepared to deal with medicine disasters. This study aimed to assess the level of knowledge, attitude, and readiness to practice disaster medicine among health care workers in the United Arab Emirates (UAE) and determine the influence of sociodemographic factors on the practice of disaster medicine. A cross-sectional survey conducted among various healthcare professionals in different healthcare facilities in the UAE. An electronic questionnaire was used and randomly distributed throughout the country. Data were collected from March to July 2021. The questionnaire consisted of 53 questions distributed among four sections: demographic information, knowledge, attitude and readiness to practice. The questionnaire distribution involved a 5-item of demographic information, a 21-item of knowledge, a 16-item of attitude and an 11-item of practice. A total of 307 (participation rate ~80.0%, n = 383) health professionals practicing in the UAE responded. Of these, 191 (62.2%) were pharmacists, 52 (15.9%) were physicians, 17 (5.5%) were dentists, 32 (10.4%) were nurses, and 15 (4.9%) were others. The mean experience was 10.9 years [SD ±7.6] (median 10, IQR 4–15). The median (IQR) overall knowledge level was 12 (8–16) and the maximum knowledge level was 21. The overall knowledge level differed significantly between the age groups of the participants (p = 0.002). The median (IQR) of overall attitude was (57, 50–64) for pharmacists, (55, 48–64) for physicians, (64, 44–68) for dentists, (64, 58–67) for nurses, and (60, 48–69) for others. The total attitude score differed significantly between the different professional groups (p = 0.034), gender (p = 0.008) and workplace (p = 0.011). In terms of readiness to practice, respondents’ scores were high and not significantly related to age (p = 0.14), gender (p = 0.064), professional groups (p = 0.0.762), and workplace (p = 0.149). This study showed that health professionals in the UAE have moderate levels of knowledge, positive attitudes, and high readiness to engage in disaster management. Gender and place of work can be considered as influencing factors. Professional training courses and educational curriculums related to disaster medicine can be beneficial to further reduce the knowledge-attitude gap.

## Introduction

The rapid urbanization and global population growth have increased the number of natural and manmade disasters that can occur on a daily basis [1]. Many of the disasters are accelerated due to global warming and a change in the natural environment. The World Health Organization (WHO) defines a disaster as ‘a serious disruption of the functioning of a community or a society causing widespread human, material, economic or environmental losses that exceed the ability of the affected community or society to cope using its resources’ [2]. Disasters can be considered an outcome of the risks involved in any process due to multiple hazards and insufficient preparedness to prevent their occurrences [2]. According to the Emergency Events Database (EM-DAT), disasters are divided into *natural calamities* such as geological disasters (earthquakes), meteorological disasters (cold waves), hydrological disasters (i.e. floods), climatological disasters (lake outburst), etc. and *catastrophic events* caused by anthropogenic activity such as industrial accidents (chemical spill), transport accidents and miscellaneous accidents such as an explosion [3]. From 1970 to 2016, the number of catastrophic events has increased while in 2017, there has been a reduction in the number of events [4]. This reduction is attributed to the early warning systems; however, frequent changes in weather events, urbanization, and increasing population growth in high-risk areas increase the exposure of people to catastrophes [5]. The outcome of disasters varied from loss of lives to economic effects [6, 7]. Crisis/disasters are considered a function of risk as they cause a distraction to society and losses at different levels; material, economic, or environmental [8]. There are different consequences, which include high costs over time, great economic and political impacts, social and psychological disorders, destruction of infrastructure [9–12].

Previous research evaluated healthcare providers’ understanding and preparedness for medicine disaster management. One study from Malaysia (2016) evaluated nursing and medical professionals’ knowledge, attitude, and practice working in the emergency department and showed respondents to have a positive attitude and enough knowledge about medicine disasters [13]. Another study conducted in Saudi Arabia (2021) found that the pharmacist roles during emergencies were unclear, and a guide is needed to enhance and support the preparedness of healthcare facilities [14]. On the other hand, a recent study in Pakistan (2022) illustrated that healthcare professionals had an ‘average’ overall knowledge, attitude, and readiness for the practice of disaster management [15]. In UAE, a study (2014) showed that the country has an advanced care system and training program related to disaster management [16]. Another recent study (2021) in UAE showed that more efforts are needed to control the barriers affecting willingness to work in disasters [17].

The United Arab Emirates (UAE) is vulnerable to different natural dangers, including those atmospheric, geological, and human beings. This is due to an increase in the population, and a rise in building density without a clear hazard planning policy [18]. These risks that happened in the previous 20 years are heavy rain, floods, sand storm, fog, and landslides [19]. Such situations were quite dangerous for many citizens because they were not prepared. In April 2015, a severe dust storm hit the Middle East, which caused extremely low visibility and resulted in hundreds of road accidents, dozens of flight delays, and school closures [20].

In UAE, emergency medicine has developed very fast in a short period of time. In 2007, the National Crisis and Emergency Disaster Management Authority (NCEMA) was established to regulate and coordinate all stages of dealing with emergency events, from the crisis to recovery from it [21]. The UAE was the first in the region to manage emergencies and has made great strides in dealing with disasters. In 2012, NCEMA held the Third Crisis and Emergency Management Conference in Abu Dhabi [21]. As a result of this conference and under the supervision of NCEMA, emergency management teams have been established in each emirate in the country to increase the preparedness and response to the crisis [22]. The COVID-19 pandemic in the last two years demonstrates the need for greater investment and readiness to deal with numerous disaster situations. According to Global Response to Infectious Diseases Index, the UAE management’s response to dealing with this epidemic was proactive [23]. The government adopted strategies and protective actions to contain the outbreak successfully. Mandatory use of face masks, social distancing, closure of the country’s borders, online education, working remotely, lockdown and limitation of public movement are all measures taken by the government to deal with such emergency events [24].

Just as disasters can happen suddenly without warning, prior planning and training in dealing with such situations would help mitigate the severity of these crises and their effects [25]. Building a workforce capable of dealing with various calamities enhances the speed of response to crises with minimal losses. Their knowledge, attitude, and readiness to practice must be assessed to show the need to train healthcare personnel to deal with disasters. Up to this date, the number of available studies that evaluate healthcare professionals’ perspectives on disaster medicine preparedness issues is limited. In addition, there is a paucity of published reports regarding this topic. Therefore, based on the importance of this topic and the rapid development the country has undergone, this study aims to assess the level of knowledge, attitude, and readiness to practice disaster medicine and preparedness among healthcare personnel in UAE.

## Methods

### Study design

This study was a cross-sectional survey conducted among diverse healthcare professionals in different healthcare settings all over the country. The questionnaire was distributed electronically using the google survey. The CHERRIES Checklist [26] was used to build up the study design and write-up. Each participant can respond to the survey only one time as they have to sign in by email to start the survey. All items in the questionnaire must be answered and the responses will not be submitted if any item is missing. Data were collected from 1^st^ March to 31^st^ July 2021.

### Ethical considerations

The study had been approved by Ajman University Research Ethics Committee (P-H-F-Feb 13, Approval Date: February 18th, 2021). The consent form was included with the survey circulated to participants. The participants first had to read and consent to the survey prior to proceeding to fill the survey. Answering the survey meant that respondents agreed to participate in the study. The data collected was treated confidentially, and the participants were informed that they could withdraw from the study at any time. The questionnaire was anonymous, and the researchers used only codes in the analysis. The authors followed all the guidelines set by the ethical approval body.

### Study participants and sample size

Healthcare professionals from different hospitals, primary health care centers, GP clinics and community pharmacies all over the country were invited to participate in the study. The sample size has been calculated using modified Cochran Formula. According to UAE statistics, the estimated number of healthcare personnel is approximately 113000. Using the above-mentioned equation and considering the margin of error of 5% (CI = 95%), the response distribution of 50%, the minimum sample size required was 383.

### Sampling methods

A stratified simple random sampling technique was used where the total number of samples were divided into seven emirates based on the percentage of healthcare personnel in each emirate.

### Inclusion and exclusion criteria

Any healthcare personnel working in hospitals, primary health care centers, GP clinics, or community pharmacies were invited to participate in the study. Other service workers in the health sectors were excluded from the study.

### Study tool

In this research, the authors chose a pre-validated questionnaire used by other researchers in Qatar with their permission [27]. The was further revised when a study was carried out in Pakistan among healthcare professionals [15]. The study tool had 3 sections (knowledge, attitude, and readiness to practice) in addition to participants’ demography. Participants’ KAP levels were defined as ‘low’, ‘moderate’ and ‘high’ based on Bloom’s cutoff point [28].

*Section 1 (knowledge)* had 21 ‘yes/no’ questions with one point for each question. Responses with scores < 6 points were categorized as ‘low’, 6–16 points ‘moderate’, >16 points ‘high’. *Section 2 (attitude)* is composed of 16 Likert scale questions with responses starting from ‘strongly agree’ to ‘strongly disagree’. The points ranged from 16 to 80 points. Results with scores < 42 points were grouped ‘low’, 42–56 points ‘moderate’, and > 56 points ‘high’. *Section 3 (practice readiness)* similar to the attitude section, was Likert scale questions with a total of 11 statements. Responses started by ‘strongly agree’ and ended by ‘not applicable’. Scores < 31 points were labelled ‘low’, 31–38 points ‘moderate’, and scores > 38 points ‘high’. The reliability was checked by calculating Cronbach’s alpha value after obtaining all the responses and yielded a value 0.947.

### Data collection method

A self-administered survey was conducted to collect data from the healthcare professionals. The Google survey link was distributed electronically using emails, WhatsApp, and social media platforms (Instagram, Facebook, and LinkedIn). In addition to that, a representative from different healthcare centers, clinics, hospitals and pharmacies was assigned to increase the number of participants. The surveys were distributed through I Pads and asked them to fill on the spot. Participants were selected conveniently without limitations on gender, age, or experience.

### Data analysis

The SPSS Version 28 (IBM Corp. Released 2021. IBM SPSS Statistics for Windows, Version 28.0. Armonk, NY: IBM Corp) was used to analyze the data. The questionnaire data normality was checked using the Shapiro-Wilk test resulting in a p-value less than 0.001. Descriptive analysis, frequency (%) for noncontinuous variables, and median (IQR) for continuous variables were used. For easy interpretation, the findings related to attitude and practice (Tables 2 and 4) were also presented in frequencies. Since the data were not normally distributed, nonparametric tests (i.e., Kruskal–Wallis, and Mann–Whitney) were used. Post hoc analyses were performed whenever applicable using Bonferroni correction. All tests were carried out at an a priori alpha level of 0.05.

## Results

### Demographic characteristics of the study participants

A total of 307/383 health professionals practicing in the UAE responded, for a participation rate of 80.15%. Of them, 179 (58.3%) were female and 128 (41.7%) were male. The mean age (sd) was 35.71 (9.01) with a median (IQR) of 35 (29–41). The age range of respondents less than 26 years old was 41 (13.4%), 26–50 years old was 247 (80.5%), and over 50 years old was 19 (6.2%). Of all participants, 191 (62.2%) were pharmacists, 52 (15.9%) were physicians, 17 (5.5%) were dentists, 32 (10.4%) were nurses, and 15 (4.9%) were others. The mean experience was 10.9 years [SD ±7.6] (median 10, 4–15). Regarding workplace, 125 (40.7%) participants worked in hospitals, 37 (12.1%) in primary health centers, 57 (18.6%) in community pharmacies, 21 (6.8%) in GP clinics, and 67 (21.8%) in others.

### Part A: Knowledge assessment

**Table 1** contains the median scores (IQR) for each knowledge question. The overall median (IQR) knowledge score was 12 (8–16) and the maximum knowledge score was 21.

**Table 1:** Knowledge among HCPs on medicine disasters.

Knowledge assessment	Yes (%)	No (%)
**1. I have previous exposure to this topic (*Disaster Medicines Preparedness*).**	90 (29.3)	217 (70.7)
**2. I have previous experience in dealing with disasters.**	111 (36.2)	196 (63.8)
**3. I think UAE is at risk of disasters (natural or human made).**	109 (35.5)	198 (64.50
**4. Disasters come in many shapes and sizes.**	266 (86.6)	41 (13.40
**5. Disaster medicine is the sole responsibility of pharmacy organization.**	153 (49.8)	154 (50.2)
**6. I read journal articles related to medicines disaster preparedness.**	119 (38.8)	188 (61.2)
**7. I am aware of programs about disaster medicines preparedness and management that are offered for example at either my workplace, or community.**	136 (44.3)	171 (55.7)
**8. I find that the research literature on disaster medicines preparedness and management is easily accessible.**	138 (45)	169 (55)
**9. I find that the research literature on disaster medicines preparedness is understandable.**	157 (51.1)	150 (48.9)
**10. Finding relevant information about disaster medicines preparedness related to this country needs is an obstacle to my level of preparedness.**	157 (51.1)	150 (48.9)
**11. I know where to find relevant research or information related to disaster medicines preparedness and management to fill in gaps in my knowledge.**	158 (51.5)	149 (48.5)
**12. I know referral contacts in case of a disaster medicines situation (e.g. health department).**	171 (55.7)	136 (44.30
**13. In case of a disaster medicines situation I think that there is sufficient support from local officials on the governance level.**	222 (74.3)	85 (25.7)
**14. I am aware of what the potential risks emergencies in this country are (e.g.: natural disaster, embargo, terror, war…etc.).**	200 (65.1)	107 (34.9)
**15. I know how such emergencies or disaster can affect the medication supply system (selection, quantification, procurement, storage, distribution).**	223 (72.6)	84 (27.4)
**16. I know the limits of my knowledge, skills, and readiness as a healthcare personnel to act in disaster medicines situations, and I would know when I exceed them.**	222 (72.3)	85 (27.70
**17. I am familiar with the local emergency response system for medicines disasters.**	152 (49.5)	155 (50.5)
**18. I am familiar with the accepted process of ‘examining problems in order to decide which ones are the most serious and must be dealt with first (triage principles)’ used in disaster medicines situations.**	143 (46.6)	164 (53.4)
**19. I am familiar with the organizational logistics and roles among local and national agencies in disaster medicines response (i.e. taking decisions and measures) situations.**	140 (45.6)	167 (54.4)
**20. Realistic on-scene training is vital to an efficient and effective disaster medicines plan.**	233 (75.9)	74 (24.1)
**21. Disaster medicine is truly a systems-oriented specialty, and involved multiple responding agencies.**	215 (81.4)	57 (18.6)

*Gender*: There was no statistically significant difference between males and females in knowledge score (two-sided p = 0.765, male score 12 (7–17) and female median score 12 (8–16)).

*Professions*: The mean score for pharmacists was 11.91 [SD ±4.94], for physicians 10.67 [SD ±8.40], for dentists 10.11 [SD ±4.91], for nurses 12.94 [SD ±6.63], and for others 8.93 [SD ±5.19]. There was no statistically significant difference in knowledge scores between professional groups (p = 0.107).

*Age group*: the median (IQR) of the total knowledge score for those under 26 years old is 12 (8–14), for those 26–50 years old is 12 (7–16), and for those over 50 years old is 19 (11–21). There is a statistically significant difference between age group and total knowledge score (p = 0.002).

The post-hoc Bonferroni test revealed a significant difference between those under 26 and those over 50 (p = 0.005) and those 26–50 and those over 50 (p < 0.001).

*Workplace*: the level of knowledge in the categories: Hospitals is 14 [9–17], primary healthcare is 11 [5–17], community pharmacy is 10 [7.5–16], GP clinic is 15 [2.8–18] and others are 11 [8–14], with no significant difference p = 0.184.

### Part B: Attitude assessment

**Table 2** shows the median scores (IQR) for each attitude-related question. The overall median (IQR) attitude score was 59 (49–64) and the maximum knowledge score was 80. The descriptive form of analysis of the Likert responses have been listed in Table 2.

**Table 2:** Attitude among HCPs on medicine disasters.

Attitude assessment	SDA (n,%)	DA (n,%)	N (n,%)	A (n,%)	SA (n,%)	Median (IQR)
**1. I consider myself prepared for the management of disasters medicines.**	17 (5.5)	60 (19.5)	81 (26.5)	89 (29)	60 (19.5)	3 (2–4)
**2. I would feel confident in my abilities as a healthcare personnel in disaster medicines situation.**	60 (19.5)	31 (10.1)	87 (28.3)	124 (40.4)	5 (1.6)	3 (2–4)
**3. I would be interested in educational classes on disaster medicines preparedness that relate specifically to the country situation**	8 (2.6)	11 (3.6)	40 (13)	126 (41)	122 (39.7)	4 (4–5)
**4. I would be considered a key leadership figure in my community in a disaster medicines situation.**	9 (2.9)	33 (10.7)	86 (28)	110 (35.8)	69 (22.5)	4 (3–4)
**5. I have personal/family emergency plans in place for disaster medicines situations.**	19 (8.2)	67 (21.8)	93 (30.3)	84 (27.4)	44 (14.3)	3 (2–4)
**6. I have an agreement with loved ones and family members on how to execute our personal/family emergency and disaster medicines plans.**	23 (7.5)	63 (20.5)	89 (29)	93 (30.3)	39 (12.7)	3 (2–4)
**7. I am able to describe my role in the response phase of a disaster medicines in the context of my workplace, the general public, media, and personal contacts.**	9 (2.9)	57 (18.6)	70 (22.8)	132 (40.1)	48 (15.6)	4 (3–4)
**8. I would feel confident as a future manager or coordinator of a shelter/healthcare/ medication supply facility.**	6 (2)	36 (11.7)	68 (22.1)	138 (45)	59 (19.2)	4 (3–4)
**9. I would be willing to be a future member of a healthcare response team in case of a medicines disaster.**	3 (1)	22 (7.2)	58 (18.9)	140 (45.6)	84 (27.4)	4 (3–5)
**10. I feel reasonably confident I can care for patients independently without supervision in a medicines disaster situation.**	9 (2.9)	47 (15.3)	68 (22.1)	127 (41.4)	56 (18.2)	4 (3–4)
**11. I would feel confident implementing emergency and disaster medicine plans and procedures.**	8 (2.6)	41 (30.4)	68 (22.1)	136 (44.3)	54 (17.6)	4 (3–4)
**12. I would feel confident in providing medicine-related education in case of disaster or emergency.**	11 (3.6)	30 (9.8)	61 (19.9)	140 (45.6)	65 (21.2)	4 (3–4)
**13. As a health personnel, I consider myself prepared for the management of medicines disasters.**	13 (4.2)	48 (15.6)	72 (23.5)	129 (42)	45 (14.7)	4 (3–4)
**14. As a health personnel, I would feel confident in my abilities as a future healthcare provider and first responder in medicines disaster situation.**	6 (2)	36 (11.7)	67 (21.8)	139 (45.3)	59 (19.2)	4 (3–4)
**15. There’s enough awareness on “ways to stand wars and other humanity and natural emergencies” among healthcare personnel in the workplace**	11 (3.6)	58 (18.9)	72 (23.5)	114 (37.1)	52 (16.9)	4 (3–4)
**16. I need more workshops and simulated training to be ready for dealing with disaster medicines.**	6 (2)	12 (3.9)	44 (14.3)	154 (50.2)	91 (29.6)	4 (4–5)

SA: strongly agree, A: agree, N: Neutral, D: dis agree, SDA: strongly disagree

*Gender*: the median (IQR) total attitude score was (61, 53–65) for males and (56, 48–64) for females. There was a statistically significant difference between males and females on the total attitude score (p = 0.008).

*Professions*: The median (IQR) of overall attitude was (57, 50–64) for pharmacists, (55, 48–64) for physicians, (64, 44–68) for dentists, (64, 58–67) for nurses, and (60, 48–69) for others. There was a statistically significant difference in total attitude scores between health professions groups (p = 0.034).

Post-hoc analysis (Bonferroni correction for multiple testing) revealed a statistically significant difference between physicians and nurses with a p-value of 0.003 compared to nurses and also between pharmacists and nurses, p = 0.004.

*Age group*: the influence of the age group had no statistically significant difference in the total attitude score with p = 0.067. The median for those under 26 years old was (63, 50–67), for those 26–50 years old was (58, 48–64), and for those over 50 years old was (64, 55–65).

*Workplace*: there was a statistically significant difference between workplace and total attitude score (p = 0.011). Median attitude scores (IQR) were (62, 55–66.5) for hospitals, (58, 50–64) for primary care, (55, 48–64) for pharmacies, (64, 55.5–74.5) for GP, and (56, 46–64) for others.

The post hoc analysis of the pairwise comparisons of the workplace in relation to the overall attitude scores is shown in **Table 3.**

**Table 3:** Pairwise comparisons of work place regarding total attitude score.

Pairwise work place	p values *
**Community pharmacy–Hospitals**	0.035
**Community pharmacy–GP clinic**	0.015
**Primary Healthcare–GP clinic**	0.045
Others**–Hospitals	0.01
Others**–GP clinic	0.007

*Post hoc analyses performed using Bonferroni test, **Other include primary healthcare, university clinics, Dubai health authority, regulatory affairs etc.

### Part C: Readiness to practice assessment and barriers

**Table 4** lists the readiness to practice and barriers among HCPs in medicine disasters. The overall median (IQR) readiness to practice score was 39 (33–44) and the maximum knowledge score was 40. The descriptive form of analysis of the Likert responses is provided in Table 4.

**Table 4:** Readiness to practice and barriers among HCPs on medicine disasters.

Readiness to Practice Assessment	NA	SDA (n,%)	DA (n,%)	N (n,%)	A (n,%)	SA (n,%)	Median (IQR)
**1. My role in disaster medicines situations is clear.**	14 (4.6)	19 (6.2)	38 (12.4)	83 (27)	111 (36.2)	42 (13.7)	3 (3–4)
**2. I am ready to handle whatever potential risks emergencies exist in the community.**	8 (2.6)	7 (2.3)	39 (12.7)	84 (27.4)	121 (39.4)	48 (15.6)	4 (3–4)
**3. I am willing to attend the emergency medicine education incorporated in the continuous professional education program.**	3 (1)	4 (1.3)	19 (6.2)	52 (16.9)	143 (46.6)	86 (28)	4 (3–5)
**4. I attended workshops/seminars about disaster medicine and it is enough for me to practice in real situation.**	9 (2.9)	31 (10.1)	70 (22.8)	69 (22.5)	92 (30)	36 (11.7)	3 (2–4)
**5. My college courses enable me to be ready to practice in the settings of disaster (natural: e.g.- earthquakes and floods; or human made: e.g.- embargo or wars) **	11 (3.6)	31 (10.1)	52 (16.9)	74 (24.1)	96 (31.3)	43 (14)	3 (2–4)
**6. Other extracurricular resources (e.g.: internet, TV, radio and newspapers) enables me with a sufficient degree of readiness to practice under disaster. **	8 (2.6)	16 (5.2)	44 (14.3)	83 (27)	118 (38.4)	38 (12.4)	4 (3–4)
**7. I’m ready to practice under disaster knowing that some basic medications may not be available because of the disaster situation. **	5 (1.6)	13 (4.2)	34 (11.1)	59 (19.2)	148 (48.2)	48 (15.6)	4 (3–4)
**8. I need to be more trained on providing patient-centered care under the situation of disaster medicines. **	6 (2)	4 (1.3)	18 (5.9)	46 (15)	141 (45.9)	92 (30)	4 (4–5)
**Barriers assessment**							
**The following are** **barriers** **that reduce my readiness to practice:**							
**9 a. Lack of knowledge about medications disaster.→ being unfamiliar with the new medications appearing during disasters. (The previous few questions are dealing with the same issue).**	7 (2.3)	4 (1.3)	18 (5.9)	57 (18.6)	157 (51.1)	64 (20.8)	4 (3–4)
**9b. Disasters medicines are unlikely to occur in UAE.**							
**9c. It requires effort and time to be prepared.**	14 (4.6)	8 (2.6)	48 (15.6)	111 (36.2)	101 (32.9)	25 (8.1)	3 (3–4)
	5 (1.6)	5 (1.6)	12 (3.9)	49 (16)	150 (48.9)	86 (28)	4 (4–5)

NA: not applicable, SA: strongly agree, A: agree, N: Neutral, D: dis agree, SDA: strongly disagree

*Gender*: the median (IQR) for readiness to practice was (39, 33–44) for women and (41, 33–44) for men. There was no statistically significant difference between men and women (p = 0.064) using the Mann-Whitney U test.

*Professions*: The median (IQR) total practice readiness score was 39 (34–44) for pharmacists, 38.5 (33–44) for physicians, 39 (31.5–44) for dentists, 42 (33–45) for nurses, and 41 (28–46) for others. There was no statistically significant difference between the profession groups (p = 0.0.762).

*Age group*: the median (IQR) of the total score for readiness to practice was (42, 34–44) for those under 26 years old, (39, 33–44) for those 26–50 years old, and (44, 38–44) for those over 50 years old. There was no statistically significant difference between the age group and total practice readiness score (p = 0.14).

*Workplace*: median (IQR) total practice readiness score was (39, 34–44) for hospitals, (39, 33–44) for primary care, (39, 33–43) for community pharmacies, (43, 32.5–49) for clinic GP, and (38, 32–42) for others, with no significant difference (p = 0.149).

## Discussion

This nationwide survey assessed the healthcare personnel’s knowledge, attitude, and readiness to practice disaster management in different emirates all over the UAE. The findings shows that healthcare providers have ‘moderate’ knowledge levels about medicine disasters, but they have different attitudes depending on their place of work in dealing with crises.

Recently, the world has perceived an increase in disasters that threaten the health status of human beings, e.x, the floods hit UAE in July, 2022 where seven people dead [29]. Because of the negative impact that disasters have on different levels; social, environmental, and economic, the WHO encouraged countries to have active plans in place to deal with these events [30]. Healthcare professionals are expected to have the required level of competencies and training at all levels to contribute in readiness, recovery, and relief during emergencies and crises as recommended by the Joint Commission on Accreditation of Healthcare Organizations (JCAHO) [31]. A well-prepared healthcare system can contribute a lot to alleviating the human suffering caused due to disasters [25] through quicker response, proper coordination, and effective utilization of available resources.

The results showed no significant difference between males and females in the knowledge score. This result is different from the one shown by another study conducted in Pakistan in 2022, where the females have a higher level of knowledge about medical disasters than males [15]. In contrast, a study conducted in Australia (2011) showed that males have better knowledge than females [32]. This diversity in the knowledge level between males and females may be due to the number of participants’ gender in each study, while in our study, the percentage of males and females who have participated are close.

In the present research, knowledge levels among health care professionals were moderate. This outcome is compatible with other research. An example of these studies was the one conducted in Pakistan, Gillani et al. concluded that physicians, pharmacists, dentists, and nurses had moderate knowledge levels about disaster management [15]. Another Saudi Arabian study reported a satisfactory level of knowledge among physicians and nurses [33]. One study from Iran (2020) found that nurses had a ‘moderate’ level of knowledge [34]. Osman (2016) found that healthcare providers working in emergencies had adequate knowledge of disaster management [13]. In opposition to these studies, two previous studies conducted in Malaysia (2012) [35] and another one in Australia (2011) [32] indicated that less than half of the participants had a low level of knowledge. In order to raise knowledge level among healthcare providers, training courses and CME conferences can be held and the topic of medicine disaster to be included in undergraduate curriculums.

Another element that could affect knowledge level is age. Ppresent research found that participant’s age affects knowledge level significantly. This may be due to having more experience over the passing years and working in different healthcare settings. On the other hand, there were no significant difference between knowledge and the working place of healthcare providers was seen. Overall, these studies verified that the healthcare staff has inadequate levels of knowledge. Findings of this research thus suggests that healthprofessionals under study may not be able to deal with emergency events professionally in the future without undergoing trainings. To raise their knowledge level, more training courses in different disasters have to be done and conferences related to disaster medicines are urgently needed.

Regarding the attitude of healthcare professionals, our findings indicated a positive attitude among healthcare providers. This shows that if one can improve the knowledge, curriculums, training courses, and awareness conferences, then, healthcare professionals are willing to be involved in managing disaster events. Moreover, participants’ gender, professional groups, and workplace are significantly related to attitude. The nurses, dentists, and pharmacists showed a high attitude toward disaster medicine management, while physicians had a moderate attitude. This result was similar to the one conducted in Yemen (2018) where the healthcare personnel showed a positive attitude [36]. Likewise, other studies conducted in Malaysia (2012, 2016) showed the good attitude of participants towards disaster preparedness [13, 22]. However, Gillani—et al. (2022) discovered that the attitude of healthcare professionals was low [14]. Similar to it, Diab and Mabrouk (2015) found that only 37.5% of them had a positive attitude [37]. Another study in Saudi Arabia (2018) showed a neutral attitude of the healthcare providers [33]. In Iran, a study conducted by Far et al. discovered that nurses had a ‘moderate’ attitude towards disasters [34]. All these studies illustrated participants’ low to moderate attitude levels towards disasters. Therefore, the educator institutes and healthcare institutions should adopt curriculums and policies to change the way of thinking of healthcare providers about medicine disasters.

Other key findings of the primary data are that the respondents’ readiness levels were high, and there was no significant relationship between participants’ age, gender, professional groups, working place, and readiness to practice. This means that the problem is not at the individual level, but more at the management level and systems have to be established to be followed during disasters. This result contrasts with previous studies that showed moderate to low levels of healthcare professionals’ readiness to practice medicine management. Gillani—et al. showed that the medical staff working in hospitals practiced disaster management moderately [15]. Nofal et al mentioned the low practice level of emergency staff towards disasters [33]. Another study conducted by Far et al. discovered that nurses had moderate performance related to disaster management. Other factors such as gender, age, marital status, and job experience can affect their readiness significantly [34]. In Malaysia (2012), a study found that nurses had adequate practice levels [35]. In another study in South Africa, the results showed that the participants’ practice was probably inadequate [38]. Overall, these studies illustrated a moderate level of readiness to practice among the healthcare staff.

### Study limitations

This study has a few limitations. Firstly, the survey response rate was only 80.15% and hence the results of this study cannot be generalized to all workers in health sectors. Due to the COVID-19 pandemic, there is a possibility that the knowledge, attitude, and readiness of the respondents could have been high. Since the study was based on a self-reported survey, the Dunning-Kruger effect (i.e. cognitive bias) and social desirability factor are possible and cannot be ignored.

### Recommendations

Based on the research findings, the authors recommend that colleges and universities consider including theoretical and practical courses on disaster management in their curricula. Continuous education programs related to medicine disasters for healthcare professionals should be conducted regularly. Healthcare institutions’ administrators must ensure personnel capacity and resources availability during catastrophic events. Since disaster management is multidisciplinary teamwork, strategies for interprofessional collaboration between diverse health workers during disasters should be in place to effectively handle the disasters. In addition, interprofessional education should be implemented at the college level to nurture the culture of working together as future health professionals. It is also important to prepare future generations for more challenges.

## Conclusion

This study demonstrated that the UAE healthcare professionals have a moderate level of knowledge, a positive attitude, and a high readiness to practice disaster management regardless of their professional background. Findings suggest a potential role and scope for multidisciplinary healthcare team in handling medicine disasters if trained well. Development of professional training courses and educational curriculums related to medicine disasters can reduce the gap between knowledge and attitude of healthcare professionals towards disasters events. Further studies are required to confirm the contribution of these factors to disaster management knowledge, attitude, and practice.

### Questionnaire permission

A special thanks to authors of the article ‘Gillani AH, Mohamed Ibrahim MI, Akbar J, Fang Y. Evaluation of Disaster Medicine Preparedness among Healthcare Profession Students: A Cross-Sectional Study in Pakistan. Int J Environ Res Public Health. 2020 Mar 19;17(6):2027’ for permitting us to use the survey tool. This tool was further revised by the original developer in the researcher ‘Gillani AH, Li S, Akbar J, Omer S, Fatima B, Ibrahim MIM, Fang Y. How Prepared Are the Health Care Professionals for Disaster Medicine Management? An Insight from Pakistan. Int J Environ Res Public Health. 2021 Dec 25;19(1):200’ with specific focus on health professionals. The senior author *(MIMI)* is part of this study in UAE.

## Supporting information

S1 Data (SAV)
